# Detection of Several Homologous MicroRNAs by a Single Smart Probe System Consisting of Linear Nucleic Acid Blockers

**DOI:** 10.3390/molecules24203691

**Published:** 2019-10-14

**Authors:** Sulayman A. Oladepo, Basiru O. Yusuf

**Affiliations:** Department of Chemistry, King Fahd University of Petroleum and Minerals, Dhahran 31261, Saudi Arabia; g201513450@kfupm.edu.sa

**Keywords:** homologous microRNAs, universal smart probe, let-7 microRNAs, mixed-base nucleic acids, linear nucleic acid blockers, LNABs

## Abstract

We report a universal smart probe (SP) that is capable of detecting several homologous let-7 microRNAs (miRNAs). While the SP is complementary to let-7a, and therefore, strongly binds to this target, due to sequence homology, the SP also has equal propensity to non-specifically hybridize with let-7b and let-7c, which are homologous to let-7a. The fluorescence signal of the SP was switched off in the absence of any homologous member target, but the signal was switched on when any of the three homologous members was present. With the assistance of nucleic acid blockers (NABs), this SP system can discriminate between homologous miRNAs. We show that the SP can discriminate between let-7a and the other two sequences by using linear NABs (LNABs) to block non-specific interactions between the SP and these sequences. We also found that LNABs used do not cross-react with the let-7a target due to the low LNABs:SP molar ratio of 6:1 used. Overall, this SP represents a universal probe for the recognition of a homologous miRNA family. The assay is sensitive, providing a detection limit of 6 fmol. The approach is simple, fast, usable at room temperature, and represents a general platform for the in vitro detection of homologous microRNAs by a single fluorescent hairpin probe.

## 1. Introduction

Smart probes (SPs) are singly-labelled fluorescent probes used for the sequence-specific recognition of nucleic acid targets [1,2,3,4,5]. They possess stem-loop, hairpin conformation, with a fluorescent dye on one end of the oligonucleotide sequence and guanine bases on the other. The guanine bases act as quenchers for the fluorophore when in close proximity. Due to its low oxidation potential, guanine effects fluorescence quenching via photoinduced intramolecular electron transfer when in contact with, or in close proximity to, the fluorophore [2,3,4,6,7]. The loop sequence of a SP is designed to be complementary to the sequence of the nucleic acid of interest, while the stem consists of self-complementary strands of about six base pairs. In the absence of the target nucleic acid, the SP exists as a hairpin, whereby the fluorescent label and the guanine quenchers are close to one another and the fluorescence signal is effectively quenched. However, when the perfectly complementary target is present, the SP spontaneously hybridizes with this target, thereby separating the fluorophore and the quencher, and thus, turning on fluorescence signal. If a nucleic acid sequence has a single-base mutation in it, the SP can differentiate between this mismatch and the target sequence by providing a relatively lower fluorescence signal compared to that of the perfect target, which indicates a less stable hybrid between the SP and the mismatch sequence. This exquisite signaling property makes SPs effective analytical tools in various applications, such as monitoring polymerase chain reactions (PCR), DNA polymerase fidelity, T4 polynucleotide kinase activity, adenosine triphosphate (ATP) detection, detection of UV-induced nucleic acid photodamage, genetic testing, and biomedical diagnostics [8,9,10,11,12,13]. SPs are structurally similar to molecular beacons (MBs), except that guanine bases are used in SPs to replace an extrinsic quencher label used in MBs. Due to certain limitations inherent to the use of MBs, it is more advantageous to use SPs [14,15]. In any event, both SPs and MBs may be used for similar applications.

MicroRNAs (miRNA) are short, non-coding ribonucleic acid strands that regulate several processes in biology [16,17]. They contribute to the initiation and progression of various diseases, such as viral infections, neurological diseases, and cancer, and miRNA levels have been shown to correlate well with such diseased states [17,18,19]. As a result, miRNAs constitute an important class of therapeutic and diagnostic (theranostic) biomarkers [18,19,20]. Specifically, the let-7 family of miRNA has been implicated in breast and lung cancers, and their levels may be used as a diagnostic tool for detection [18,19,20,21,22,23]. Given this fact, accurate detection of members of the let-7a,b,c homologous miRNA family is crucial to the early detection and prognosis of these types of cancer. Several methods have been used in the detection of miRNAs. These methods include PCR-based methods, in situ hybridization, microarray methods, and northern blotting [18,20,24,25,26,27]. Other methods include electrochemical, colorimetric, spectroscopic, and nucleic acid amplification techniques [28,29,30,31,32,33,34,35]. Although they are selective, PCR-based techniques require a purified RNA sample and can be time- and labor-intensive [36,37]. Northern blotting presents low sensitivity, requires a large amount of sample, and is cumbersome [18,20,22]. Microarray methods have the advantage of high throughput, but they have low sensitivity and specificity [18,20,37]. The electrochemical, colorimetric, and spectroscopic techniques reported so far for miRNA detection are complicated, with procedures that are rather involved, thereby making them less attractive for routine miRNA detection [27,28,29,38]. Nucleic acid amplification methods are sensitive, but they come with complex reaction or hybridization mixtures, and unintended mismatch nucleic acids may also be amplified due to non-specific hybridization, therefore presenting high levels of background signals [32,39,40]. Thus, mismatch nucleic acid sequences may present a challenge, even with nucleic acid amplification methods that are very sensitive. In addition to all of these limitations, all the methods reported so far for miRNA detection involve a single probe for a single target miRNA sequence; none of the methods so far reported has used a single probe for multiple miRNA sequences.

It may be desirable to use a single probe for the recognition of several homologous, mixed-base target sequences. This is relevant in situations where multiple targets are to be simultaneously detected, especially where several members of the same homologous family code for the same type of cancer. For example, let-7a, -7b, and -7c have been implicated in the same type of cancer [21,23]. Non-specific interactions between the probe and unintended (mismatch) sequences can in fact be exploited for good use in miRNA detection. Thus, a probe can interact with multiple sequences via specific and non-specific hybridizations, making the probe universal for detecting multiple sequences. Such a universal probe system is desirable and would be a good addition to the repertoire of tools available to scientists for cancer biomarker detection. Furthermore, when only one member of the homologous family is to be detected to the exclusion of the other members [22], the universal probe system can be conditioned such that the same probe will sequence-specifically recognize the target of interest, to the exclusion of other, unintended homologous members.

Homogeneous methods that based on SPs are suitable for miRNA detection [38,40]. They are simple and fast, and present good sensitivity and specificity. In the presence of single-base mutations, non-specific interaction between mismatch sequences and the SP may be significant, especially if mixed-base sequences are involved [40]. The specificity of SPs in the face of non-specific interactions and sequence homology can be significantly improved by making use of nucleic acid blockers (NABs) [40]. These are unlabeled nucleic acids that are complementary and specific to the mismatch sequence of interest. They prevent non-specific interactions between the probe and unintended nucleic acid (mismatch) sequences by specifically hybridizing with such mismatch sequences, thereby isolating the SP to hybridize exclusively with the target sequence of interest. Oligonucleotide blockers have been previously used in the detection of single nucleotide mutation [41,42], and our research group recently reported a simple protocol involving the use of hairpin-shaped NABs for mixed-base miRNA detection [40]. Thus, unlike other methods that have so far been used for miRNA detection, homogeneous methods based on the SP/NABs system can offer good sensitivity, while also presenting excellent discrimination between the miRNA target of interest and similar mismatch sequences, including single nucleotide polymorphism (SNP).

In the work being presented here, we designed and characterized a universal SP detection system for the recognition of let-7a, -7b, and -7c, which are homologous members of the same miRNA family, and are biomarkers for breast and lung cancers. The SP was designed to be strictly complementary to let-7a, and therefore, would hybridize with this target sequence-specifically. However, due to the sequence similarity between let-7a, let-7b, and let-7c, non-specific interaction between the SP and let-7b and let-7c is also significant. Therefore, the SP is expected to hybridize with all three sequences, i.e., let-7a,b,c, with essentially equal propensity. This single SP thus offers a universal detection for all three homologous sequences. In addition, for sequence-specific recognition of let-7a alone, without interference from homologous let-7b and let-7c, we introduced linear nucleic acid blockers (LNABs) to the medium to screen out possible interference due to non-specific interactions between the SP and let-7b and let-7c sequences. LNABs are non-fluorescent, linear oligonucleotides that are perfectly complementary to unintended mismatch sequences, and thereby, specifically hybridize with such sequences. The SP/LNABs system thus constitutes a universal detection system that is capable of simultaneous recognition of all three Let-7a, -7b, and -7c homologous members; furthermore, in the presence of the appropriate LNABs, the system is also capable of discriminating between the three sequences. To our knowledge, this is the first report on the use of a single SP system for the universal detection of several homologous miRNA sequences, which also provides exquisite discrimination amongst the sequences when required. This detection system is simple and fast, it does not involve a complicated hybridization procedure, it operates at room temperature, and no washing steps are required. Furthermore, this universal detection system presents good sensitivity and sequence-specific discrimination in vitro that is better than previously-reported miRNA detection methods. We wish to state that this work involves the use of linear NABs (LNABs), whose performance and blocking characteristics may be quite different from hairpin-shaped NABs [40,41].

## 2. Results and Discussion

### 2.1. The SP and Target Sequences

**Figure 1:**
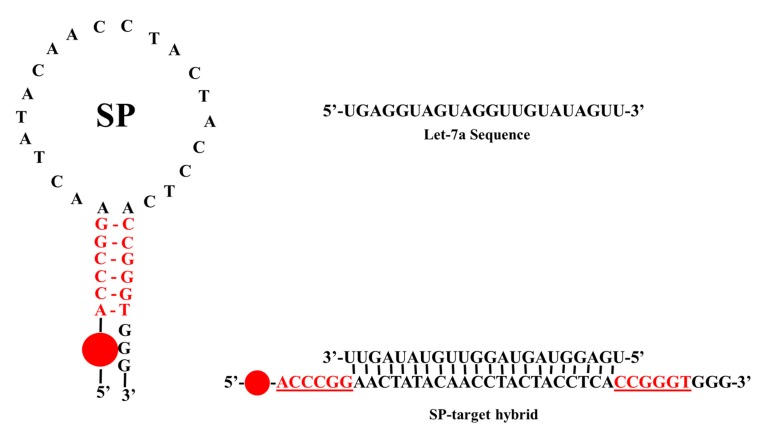
Structure of the SP and that of SP-target hybrid. The SP-target hybrid is shown in the bottom right (bases of the self-complementary stem are shown in red and are underlined). Let-7a (L7a) target sequence is also shown in the top right panel.

Table 1 presents a list of all oligonucleotides used in this work. The label shown for each nucleic acid in the right column of the table is hereby adopted for the rest of this paper. As shown in the Table, L7a, -7b, and -7c are very similar sequences, and are homologous members of the same miRNA family. The structure of the SP used in this work is shown in Figure 1. It has been carefully designed for optimal performance and for the sequence-specific recognition of L7a target. This same SP also strongly hybridizes with L7b and L7c via non-specific interaction *(vide infra)*. It consists of a total of 37 bases. The 22 bases in the loop are perfectly complementary to the L7a target sequence, while the stem consists of six self-complementary base pairs. The 5′ end of the SP is labelled with 6-carboxyfluorescein (6-FAM) fluorophore, while the 3′ end is terminated with three overhanging guanosine residues that are not part of the self-complementary stem, but act as quenchers for the fluorophore. The quenching is enhanced by three other G bases further up the strand on the 3′ side of the stem (Figure 1). These internal guanosine residues are separated from the overhanging ones by a thymine base so as to avoid the formation of G-tetraplexes and to prevent unnecessary difficulties in synthesis [38,43]. For clarity, we wish to point out that the three overhanging G’s at the 3′ end of the SP are the main guanosine quenchers that were used to quench the fluorescence signal generated by the fluorophore attached to the 5′ end of the probe. This design approach has been reported previously and has shown very good results for SP-based detections [4,11,38]. As shown in Figure 1, we have placed three more guanosine residues further up the 3′ side of the stem to further enhance fluorescence quenching. The L7a target sequence is also shown in the top right panel of Figure 1, while the SP-target hybrid is shown in the bottom right, with the fluorophore and G quencher now being far away from each other.

### 2.2. Melting Profiles

**Figure 2:**
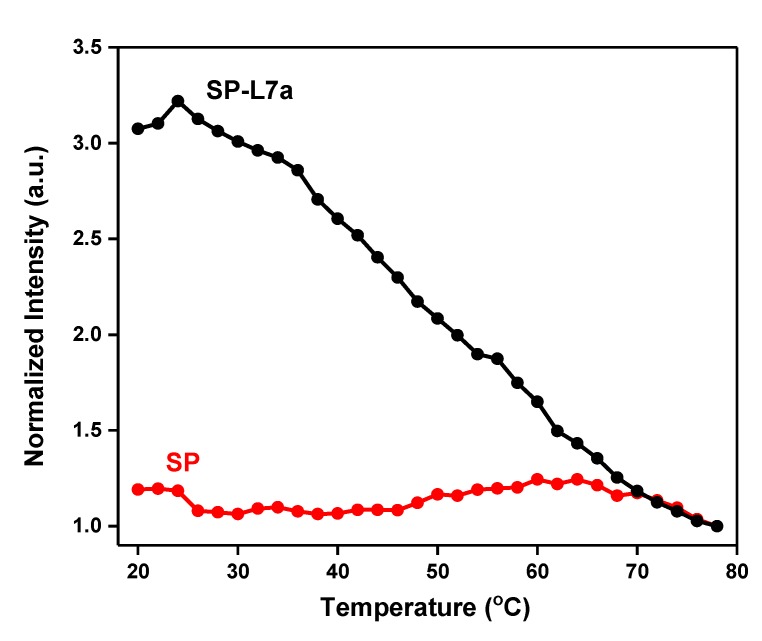
Melting profiles of SP in the absence and presence of L7a target. The corresponding raw fluorescence spectra are presented in Figures S1 and S2 of the Supplementary Materials.

Figure 2 shows the melting profiles of the SP and the SP-L7a hybrid. These profiles were obtained in each case from the temperature-dependent fluorescence emission spectra by plotting fluorescence intensity at 520 nm vs. temperature. The melting temperature (T_m_) of the SP, as determined from the inflection region of the melting profile, was found to be 58 °C, while that of SP-target hybrid is 48 °C. The thermodynamic requirements of a stable and functional hairpin probe dictate that the stem of the SP must be more thermally stable than the SP-target hybrids [15]. We have ensured this relative thermal stability by making sure the T_m_ of the stem (SP alone) is far above room temperature, and at the same time, about 10 °C higher than the T_m_ of the SP-target hybrid (Figure 1). This condition ensures that the SP exists in hairpin conformation at high temperatures when the SP-target hybrid would have melted and separated into the SP and free target.

As shown in Figure 2, in the absence of the L7a target, the SP is in the off-state, whereby at low temperatures, fluorescence is effectively switched off or quenched because the fluorophore and quencher (G bases) are in close proximity to one another (Figure 2, red curve). As the temperature is gradually increased, the stem starts to melt at around 50 °C and the fluorophore and quencher gradually separate from each other, giving rise to a stronger fluorescence signal. When the stem strands are completely unwound, the SP assumes a random-coil conformation, at which temperature the signal may slightly go down due to the fluorophore and quencher moving close to each other again, as a result of the new random-coil conformation of the SP. When a 3-fold excess L7a target is present, the SP spontaneously hybridizes with this target, separating the fluorophore and quencher and turning on fluorescence signal (Figure 2, black curve). Having the L7a target in large excess and allowing the incubation to last about 5 h ensure that all the SP molecules are completely hybridized with the target, and therefore, generate the maximum possible fluorescence signal. When the temperature is increased, the SP-target hybrid begins to melt, and the fluorescence signal gradually decreases to signify that the hybrid is becoming destabilized due to gradual melting. This decrease in fluorescence continues until the L7a target is fully detached from the SP, when the signal tends to plateau beyond 70 °C. At room temperature, the signal of the SP-L7a hybrid is about 250% relative to that of SP alone. This shows that the SP can switch on and off depending on whether the L7a target is present or not. This large signal difference at room temperature shows that this SP-based system can be used for detection at room temperature (Figure 2). The relative signals recorded here are comparable to those of nucleic acid amplification methods, where a signal generated by mismatch sequences can be about 50% that of the perfect target [33,39].

**Figure 3:**
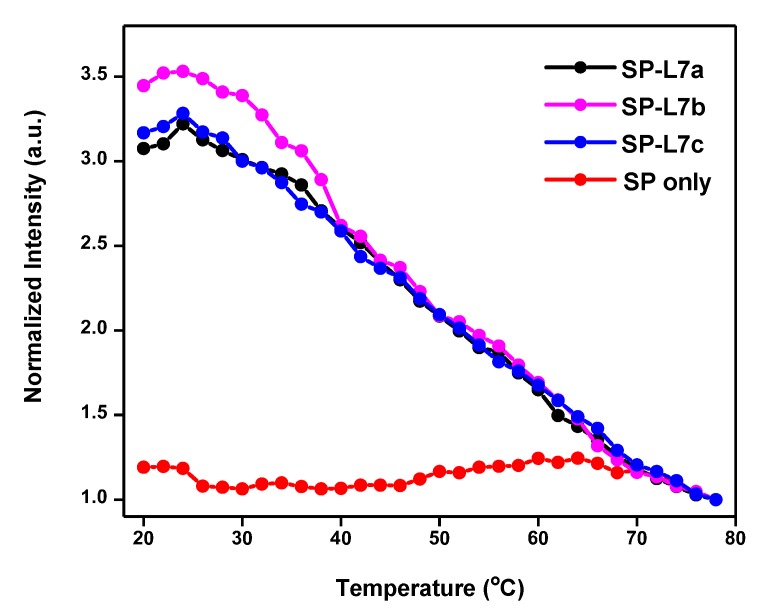
Melting profiles of SP in the presence of L7b and L7c homologous sequences. The corresponding profiles for the SP alone and SP-L7a hybrid are also shown for comparison. All the hybrids produce a high fluorescence signal, i.e., about 250% times that of SP alone. The corresponding raw fluorescence spectra for SP-L7b and SP-L7c can be found in Figures S3 and S4 of the Supplementary Materials.

The T_m_ value of the SP alone relative to that of SP-L7a hybrid gives a T_m_ difference (∆T_m_) of 10 °C. This ∆T_m_ of 10 °C suggests that the SP is more stable than the SP-target hybrid, and the thermodynamic stability requirement has been met for this SP to recognize L7a target sequence. Similar results obtained for L7b and L7c also show that this SP meets the thermodynamic stability requirement for the recognition of these homologous sequences (Figure 3). Thus, these results show that the SP satisfies the required performance characteristics both in thermal stability and target recognition.

### 2.3. Recognition of L7b and L7c through Non-Specific Hybridization

The melting profile shown in Figure 2 for SP-L7a clearly shows that L7a can switch on the fluorescence signal of the SP, thereby suggesting that the SP can recognize this target in solution. When the SP was mixed with L7b or L7c, which have double-base and single-base mismatches, respectively, with respect to L7a, the resulting hybrids also switched on the fluorescence, and their intensities are similar to that of the SP-L7a hybrid (Figure 3). This suggests that just like L7a, L7b and L7c sequences also hybridize and form stable hybrids with the SP. This means that these two sequences exhibit non-specific hybridization with the SP, while the overlapping melting curves of all the three hybrids in Figure 3 suggest that the SP can equally hybridize and, therefore, recognize all three homologous sequences. This also implies that the SP can be used to simultaneously detect all the three sequences. This observation is impressive, and highlights the possibility of using a single SP for the recognition of several homologous miRNAs. Given the location of the base mismatches in L7b and L7c sequences (Table 1), we had anticipated that the SP might bind to these sequences non-specifically, and therefore, constitute a universal probe for several miRNAs. These results confirmed our expectation. We determined the T_m_ for the SP-L7b and SP-L7c hybrids from the corresponding melting profiles, and found it to also be around 48 °C for both hybrids; the same value as for the SP-L7a hybrid (Figure 2 and Figure 3). This T_m_ value further indicates that the SP can recognize all three sequences with equal propensity. Although the sequences are subtly different, the hybrids formed between the SP and any of the three homologous sequences has practically the same thermal stability, and is therefore expected to melt at around the same temperature, i.e., 48 °C. The results shown in Figure 3 are interesting and relevant because the three homologous miRNA sequences belong to the same miRNA family, and they may jointly correlate with the same type of cancer [21,23].

### 2.4. Sequence-Specificity by Means of Linear Nucleic Acid Blockers (LNABs)

**Figure 4:**
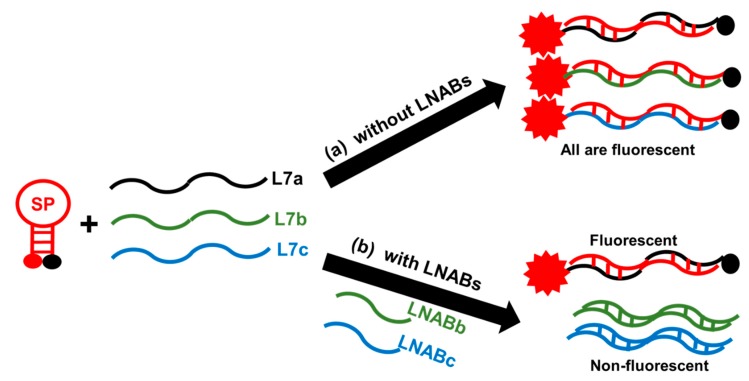
Schematic of sequence-specific hybridization between the SP and L7a, L7b, and L7c sequences in the absence and presence of LNABs. In the absence of LNABs (**a**), SP hybridizes with L7a to form SP-L7a hybrid, which is fluorescent. Non-specific interaction between the SP and homologous sequences, L7b and L7c, also gives rise to highly-fluorescent hybrids of SP-L7b and SP-L7c. When LNABs are present (**b**), only L7a specifically hybridizes with the SP to form a highly-fluorescent SP-L7a hybrid. The LNABs interact with homologous sequences, L7b and L7c, and produce non-fluorescent hybrids of L7b-LNABb and L7c-LNABc.

While it is good to be able to use a single SP for multiple nucleic acid sequences, and especially for mixed-based sequences, it may still be desirable to be able to delineate the SP for the specific recognition of only one target, with no interference from other homologous sequences. For example, one may be interested in detecting only L7a in the presence of L7b and L7c. This is especially important in situations where only this target is of interest [22], while L7b and L7c are undesirable and will give false positive results if they are present and involved in non-specific hybridization with the SP. Such a requirement means that the fluorescence signal of the SP should only be switched on in the presence of L7a; it should remain in the off state when L7b and/or L7c are present. We used LNABs to achieve such exquisite discrimination. So, in order to produce sequence-specific recognition of the L7a target by the SP to the exclusion of L7b and L7c when these sequences are present, we used LNABs to specifically hybridize with these ‘undesirable’ sequences, and therefore, prevent non-specific hybridization between them and the SP. LNABs, as used in this work, are unlabeled, non-fluorescent, linear nucleic acids (Table 1) that are perfectly complementary to L7b (LNABb) and L7c (LNABc). When these LNABs are present, they spontaneously hybridize with L7b and L7c, forming non-fluorescent L7b-LNABb and L7c-LNABc hybrids, respectively. As a result, non-specific interactions between the SP and L7b and L7c homologous sequences are thereby prevented. As shown in the scheme illustrated in Figure 4, in the absence of LNABs, the L7a target sequence and L7b and L7c homologous (mismatch) sequences all hybridize with the SP, and the fluorescence signal is switched on because of the resulting SP-L7a, SP-L7b, and SP-L7c hybrids, with all giving intense fluorescence signal. The SP hybridizes specifically with the L7a target sequence, while it engages in non-specific hybridization with L7b and L7c, also producing an intense fluorescence signal with these mismatch sequences (Figure 4a). However, when LNABb and LNABc are present, these blockers spontaneously hybridize with L7b and L7c, respectively, thereby preventing non-specific interactions with the SP; as such, the fluorescence signal remains switched off for the L7b-LNABb and L7c-LNABc hybrids (Figure 4b). By this means, the SP is spared for the exclusive hybridization with L7a, which exclusively gives an intense fluorescence signal in this case, while interference due to L7b and L7c mismatch sequences is successfully blocked.

**Figure 5:**
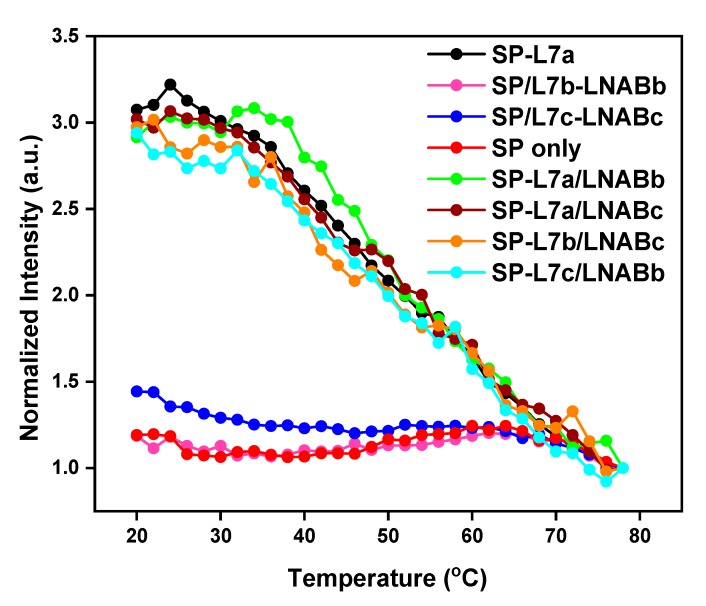
Melting profiles of hybrids of L7b-LNABb and L7c-LNABc formed between L7b and L7c in the presence of LNABs. The hybrids generate very low fluorescence signals that are essentially the inherent (background) fluorescence of unhybridized SP in hairpin conformation. The melting profile for SP-L7a is also shown for comparison. Likewise, hybrids of SP-L7a in the presence of LNABb, SP-L7a in the presence of LNABc, SP-L7b in the presence of LNABc, and SP-L7c in the presence of LNABb are shown. See Figures S1, S2, S5, S6, and S11–S14 in the Supplementary Materials for the corresponding raw fluorescence spectra.

Figure 5 shows the melting profiles of SP-L7a, as well as those of L7b and L7c in the presence of LNABb and LNABc. Specific hybridization between SP and L7a (the target of interest in this case), forms SP-L7a hybrid, which is fluorescent. Non-specific hybridization between SP and L7b and L7c homologous sequences, which is undesirable in this case, is prevented because the corresponding LNABs block such interactions. Consequently, the fluorescence signal of the SP remains switched off with respect to L7b and L7c. As shown in this figure, the signal levels when L7b, L7c, and LNABs were present are essentially reduced to that of SP alone. This means that L7b and L7c homologous sequences do not engage in non-specific hybridization with the SP whenever LNABs are present. Thus, the SP can only be switched on by L7a, i.e., the intended target sequence in this case. This is the case whether L7a is exclusively probed by the SP in an isolated solution or in the presence of L7b and L7c and their corresponding LNABs (Figure S7). Figure 5 and Figure S7 therefore present strong evidence that if one is only interested in L7a, this target can be exclusively detected by the SP, while LNABs are used to screen out non-specific interactions from L7b and L7c, thereby leaving the SP for exclusive hybridization with L7a.

We wish to emphasize that the results presented here are for a situation where L7a is the target of interest and L7b and L7c sequences are undesirable. That is why the non-specific hybridization of these homologous sequences with the SP are blocked by means of LNABb and LNABc. However, since the SP also hybridizes with L7b and L7c in the absence of LNABs, as evidenced by the observed high fluorescence signals (Figure 3), either L7b and L7c may be the main target biomarker of interest. In that case, this same universal SP may be used for this target, and sequence specificity can be achieved by using the appropriate LNABs sequences to block out the corresponding unintended homologous sequences. For instance, if L7b were the target of interest, L7a and L7c would then be the unintended mismatch sequences, and as such, they could be excluded from interacting with the SP (which binds to L7b to form SP-L7b hybrid) by using LNABa and LNABc to block out L7a and L7c accordingly. Hybrids of SP-L7b in the presence of LNABc and SP-L7c in the presence of LNABb (Figure 5) gave essentially the same fluorescence signal as SP-L7a hybrid alone, proving that, just like the L7a target, both L7b and L7c are capable of forming a highly-fluorescent hybrid with this universal SP. Thus, the SP/LNABs system used in this work is a versatile platform that can be devised for any set of homologous miRNAs. Also, due to the inherent ability of the SP to recognize homologous nucleic acid sequences, this SP-based method is expected to work for other let-7 homologous members in addition to let-7a, -7b, and -7c. We also wish to state that the SP presented in this study can also interact with various let-7 miRNA hairpin precursors [44,45]. Therefore, the SP can also be used for the recognition and detection of such let-7 miRNA hairpin precursors. In using the SP-based method for this purpose, the SP will interact with the stem regions of the hairpin precursors [16]. This further shows that the SP/LNABs system presented here has extensive utilities and versatility for probing various miRNAs.

### 2.5. No Evidence of Target-LNABs Interaction

It may be argued that when the L7a target coexists with L7b and L7c ‘mismatch’ sequences in the presence of LNABb and LNABc, non-specific interaction between L7a and LNABs is also possible. Such cross-reactivity may due to the sequence homology of L7a, L7b and L7c; if the LNABs can bind to L7b and L7c, they can equally bind to L7a. If such cross-reactivity exists and it is substantial, it will result in a decreased fluorescence signal, since the L7a target strand would bind to LNABb and LNABc. However, such a decrease in fluorescence intensity resulting from non-specific interaction between L7a and LNABs is not evident from our results. Figure 5 and Figure S7 present strong evidence that there is no cross-hybridization between L7a and LNABs, which, if present, would have lowered the signal of SP-L7a,b,c/LNABb,c shown in Figure S7, compared to that of SP-L7a of Figure 5. SP-L7a,b,c/LNABb,c, shown in Figure S7, represents SP-L7a hybrid in the presence of homologous ‘mismatch’ sequences L7b and L7c and their respective blockers, LNABb and LNABc. Our data show that the SP-L7a hybrid gave essentially the same signal intensity whether L7b, L7c, and LNABb and LNABc were present or not (black curve in Figure 5 and Figure S7). This is an indication that the L7a target does not hybridize with LNABb or LNABc to a significant extent, i.e., such that a decreased fluorescence intensity would be observed. In addition, Figure 5 also shows the hybrid of SP-L7a in the presence of LNABb, and hybrid of SP-L7a in the presence of LNABc. These hybrids gave fluorescence signals that are essentially the same as the SP-L7a hybrid (without LNABs). This again suggests that LNABs do not interfere with the SP whenever L7a, the main target sequence, is present.

It should also be noted that substantial, non-specific hybridization between L7a target and LNABs may only be possible if the SP, which is perfectly complementary to L7a, is absent. However, when the SP is present, L7a would spontaneously bind to the SP in preference to LNABs, in the same manner that the L7b and L7c homologous sequences bind to their perfectly complementary LNABs in preference to the SP as long as LNABs were present. Non-specific interaction between L7b or L7c and SP results in a substantial fluorescence signal only when LNABs are absent. So, one would similarly expect L7a/LNABs interactions to be significant only if the SP and homologous sequences (L7b and L7c) were absent, i.e., if only L7a and LNABs were present in the hybridization medium. But this is not the case here; LNABs were used when L7b and L7c mismatch sequences were present, in order to block these homologous sequences from interacting with the SP. As seen in the green and brown curves in Figure 5, even when LNABs were present without L7b and L7c sequences, LNABs still did not cross-react with L7a, because L7a preferentially hybridizes with the SP, despite the presence of LNABs. So by and large, our results do not suggest that the L7a target sequence interacts with LNABs. These results are consistent with what was previously observed for a different SP/NABs system involving a different miRNA target [40]. The observed absence of significant L7a-LNABs interaction may be rationalized on the basis of the small LNABs:SP molar ratio, 6:1, used here. A much higher molar ratio, i.e., in the region of 2000:1, could lead to reduced fluorescence signal, because the very high concentration of LNABs may mask the L7a target molecules, and therefore, prevent them from full stoichiometric hybridization with the SP [41]. In the same vein, the presence of a large excess LNABs, as obtainable with a 2000:1 LNABs:SP molar ratio, may promote non-specific hybridization between L7a and LNABs due to the fact that LNABs are present in a very large excess in comparison with SP [40]. The use of a low LNABs:SP molar ratio, i.e., 6:1, in this work precludes the masking of the target molecules by LNABs, and it does not promote non-specific LNABs/L7a interaction.

### 2.6. Concentration-Dependent Studies

**Figure 6:**
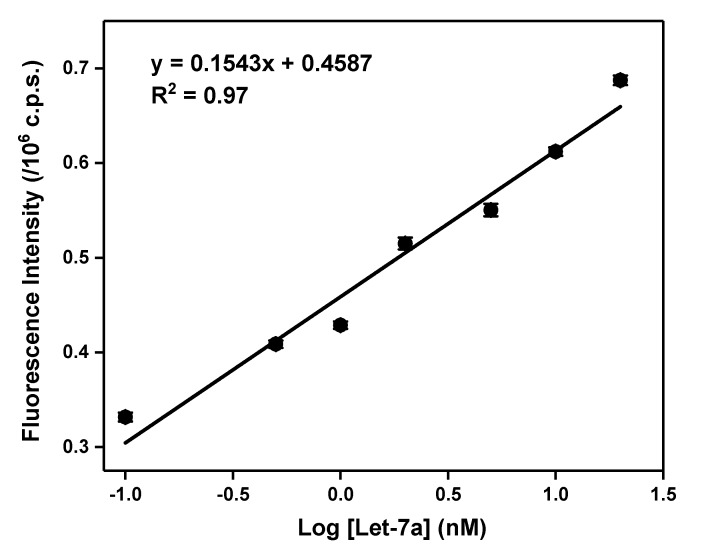
Linear section of the concentration-dependent curve for 100 nM SP mixed with varying (0–1000 nM) concentrations of L7a. Each data point is an average of three replicate measurements, and error bars representing the standard deviation are shown. The full concentration-dependent curve is shown in Figure S9, and the corresponding raw spectra are presented in Figure S10.

In order to determine the sensitivity and limit of detection of the SP for L7a target, we mixed varying concentrations of L7a with a fixed 100 nM concentration of SP. The fluorescence signal increases with L7a concentration up to about 100 nM L7a. Beyond this point, higher L7a concentrations do not result in appreciable signal increase (Figure S9). Shown in Figure 6 is the linear section of the concentration-dependent curve. We determined the limit of detection, LOD, to be 30 pM and the limit of quantitation, LOQ, to be 100 pM, while the sensitivity, which is the slope of the curve, was found to be 1.5 × 10^14^ c.p.s./M. The LOD and LOQ, respectively, represent 3 *σ*_bl_/s, and 10 *σ*_bl_/s, where *σ*_bl_ is the standard deviation of three replicate fluorescence measurements of the blank (100 nM SP without target), and s is the slope of the concentration-dependent curve. The cuvettes used in our fluorescence measurements can hold a minimal sample of about 190 μL, which means that the detection limit can reach 6 fmol in a detection volume of 200 μL. The LOD, LOQ, and sensitivity values obtained in this work are fairly superior to previous results reported for MBs and SPs in homogeneous assays [2,38,40,46]. This points to the exquisite detection sensitivity of the SP for L7a target.

## 3. Materials and Methods

### 3.1. Materials

Table 1 presents a list of the nucleic acids used in this work, including the smart probe (SP) and LNABs. Three homologous miRNAs, let-7a, let-7b, and let-7c (L7a, L7b and L7c, respectively), the SP, and LNABs were purchased from Integrated DNA Technologies, BVBA, Leuven, Belgium. All unlabeled nucleic acids were purified by standard desalting, while the SP was purified by standard desalting and HPLC. Sodium chloride was obtained from Fisher Scientific Company, Fair Lawn, NJ, USA; magnesium chloride hexahydrate (MgCl_2_·6H_2_O), ethylenediaminetetraacetic acid (EDTA), and hydrochloric acid were obtained from BDH Chemicals Limited, Poole, England; sodium hydroxide (NaOH) was purchased from Fluka AG, Buchs, Switzerland, while Tris was procured from Sigma-Aldrich, St. Louis, MO, USA. All chemicals were used as received, without further purification. All oligonucleotide solutions were prepared in nanopure water from a Barnstead Nanopure water purification system (Thermo Scientific, Waltham, MA, USA). Each oligonucleotide was dissolved in nanopure water and then stored at around −20 °C until required for experiments. When needed, each oligonucleotide solution was thawed and diluted in a buffer consisting of 20 mM Tris and 2 mM EDTA at pH 7.5, while fairly concentrated solutions of NaCl and MgCl_2_ were added to give the desired oligo concentrations in the presence of 200 mM Na and 12 mM Mg ions. UV absorbance measurements were used to confirm the oligonucleotide concentrations, as needed.

### 3.2. Absorbance and Fluorescence Measurements

We measured all fluorescence spectra on an FLS920 fluorescence spectrophotometer (Edinburgh Instruments, Livingston, UK), while UV-Vis spectra were acquired with a Genesys 10S UV-Vis spectrophotometer (Thermo Scientific, Waltham, MA, USA). These measurements were carried out as described previously [38,40]. Briefly, fluorescence measurements involving only one individual target, L7a, L7b, or L7c, made use of 100 nM SP mixed with 300 nM target (1:3 molar ratio in Tris-EDTA buffer). For all fluorescence measurements involving mixtures of targets, 50 nM SP was mixed with 150 nM concentration of L7a, L7b, and L7c (1:3:3:3 molar ratio). Whenever LNABs were used, 50 nM SP was mixed with 150 nM target in the presence of 300 nM LNABs, giving a LNABs:target molar ratio of 2:1 and LNABs:SP molar ratio of 6:1, whether the target was individually mixed with the SP or as a mixture of targets. The fluorescence spectra were acquired on 200–400 µL solutions, using a TE-cooled sample holder and a TC125 Temperature Control module (Quantum Northwest Inc., Liberty Lake, WA, USA). The temperature was programmed to vary (in 2 °C increments) from 20–78 °C, with a settling time of 100 s. In the presence of varying concentrations of L7a target sequence (0–1000 nM), 100 nM SP was used for concentration-dependent experiments. Concentration-dependent spectra were acquired at room temperature. All samples involving target sequences were incubated with the SP in the dark at room temperature for about five hours prior to fluorescence measurements unless otherwise stated. Each fluorescence spectrum was recorded at between 500–650 nm (λ_ex_ = 490 nm; λ_em_ = 520 nm). A sub-micro quartz cuvette with a 1-cm pathlength (Cole-Parmer, Vernon Hills, IL, USA) was used for all fluorescence measurements. The maximum fluorescence intensity at 520 nm was used for data analysis. Melting profiles were constructed by plotting fluorescence intensity at 520 nm vs. temperature. Melting temperature (T_m_) was determined in each case from the second derivative of the corresponding melting profile.

## 4. Conclusions

We have designed and characterized a SP for in vitro detection of let-7a, let-7b, and let-7c homologous miRNAs, which are breast and lung cancer biomarkers. The fluorescence signal of the SP switches on in the presence of any of the three homologous sequences, showing that this SP represents a universal probe for the simultaneous detection of the homologous miRNA sequences, either individually or as a mixture. When only one intended target (let-7a) was to be detected, LNABs were used to screen out unintended homologous sequences, i.e., let-7b and let-7c. So, the SP/LNABS system is also capable of providing sufficient discrimination between let-7a and the other homologous sequences of let-7b and let-7c. We found no evidence of non-specific LNABs/L7a interaction, which we ascribe to the relatively low LNABs:SP molar ratio of 6:1 used in this work. The universal SP system provides a good detection limit and sensitivity for a let-7a target. The SP used here can also be used for the recognition of various let-7 miRNA hairpin precursors, in which case the SP interacts with the stem regions of such miRNAs. The approach presented in this work can be adapted for any other homologous miRNA family. This new detection system is sensitive, selective, simple, fast, usable at room temperature, and does not involve any washing or isolation steps. It provides relative fluorescence signal levels that rival nucleic acid amplification methods, and it may be a suitable mix-and-read homogeneous platform for in vitro detection of various homologous miRNAs.

## Figures and Tables

**Table 1:** List of oligonucleotide sequences used in this work.

Sequence	Description (Label)
/56-FAM/-**ACCCGG**AACTATACAACCTACTACCTCA**CCGGGT**GGG-3′	Smart Probe (SP) ^a^
5′-rUrGrArGrGrUrArGrUrArGrGrUrUrGrUrArUrArGrUrU-3′	Let-7a (L7a)
5′-rUrGrArGrGrUrArGrUrArGrGrUrUrGrU**rG**rU**rG**rGrUrU-3′	Let-7b (L7b) ^b^
5′-rUrGrArGrGrUrArGrUrArGrGrUrUrGrUrArU**rG**rGrUrU-3′	Let-7c (L7c) ^b^
5′-AACCACACAACCTACTACCTCA-3′	Linear nucleic acid blocker for L7b (LNABb) ^c^
5′-AACCATACAACCTACTACCTCA-3′	Linear nucleic acid blocker for L7c (LNABc) ^d^

^a^ The stem strands are underlined, the three G’s on the 3′ end that are not underlined represent the quencher. ^b^ The base mismatch with respect to L7a is underlined in each case. ^c^ LNABs sequence that is perfectly complementary to L7b. ^d^ LNABs sequence that is perfectly complementary to L7c.

## Supplementary Materials

The following are available online, Figure S1: Raw fluorescence spectra of SP only (red curve in Figure 2, Figure 3 and Figure 5 of the main text and Figure S7 below). Figure S2: Raw fluorescence spectra of SP-L7a hybrid (black curve in Figure 2, Figure 3 and Figure 5 of the main text). Figure S3: Raw fluorescence spectra of SP-L7b (magenta curve in Figure 3 of the main text). Figure S4: Raw fluorescence spectra of SP-L7c (blue curve in Figure 3 of the main text). Figure S5: Raw fluorescence spectra of SP/L7b-LNABb (magenta curve in Figure 5 of the main text and Figure S7 below). Figure S6: Raw fluorescence spectra of SP/L7c-LNABc (blue curve in Figure 5 of the main text and Figure S7 below). Figure S7: Melting profiles of SP-L7a hybrid in the presence of L7b, L7c, and their corresponding nucleic acid blockers, LNABb and LNABc. SP-L7a,b,c/LNABb,c, represents SP-L7a hybrid in the presence of homologous ‘mismatch’ sequences L7b and L7c and their respective blockers, NABb and NABc. SP-L7a,b,c/LNABb represents SP-L7a hybrid in the presence of L7b and L7c and NABb, while SP-L7a,b,c/LNABc represents SP-L7a hybrid in the presence of L7b and L7c and NABc. In all cases, the signal intensity of SP-L7a,b,c, is similar to SP-L7a in Figure 2, Figure 3 and Figure 5 of the main text. This suggests little or no hybridization between L7a and NABs, while the signals of SP/L7b-NABb and SP/L7c-NABc hybrids are essentially the same as that of SP alone. Figure S8: Raw fluorescence spectra of SP-L7a hybrid in the presence of L7b, L7c, and their corresponding nucleic acid blockers, LNABb and LNABc (black curve in Figure S7). Figure S9: Raw fluorescence spectra of SP-L7a hybrid in the presence of L7b, L7c, and LNABb (green curve in Figure S7). Figure S10: Raw fluorescence spectra of SP-L7a hybrid in the presence of L7b, L7c, and LNABc (orange curve in Figure S7). Figure S11: Raw fluorescence spectra of SP-L7a hybrid in the presence of LNABb (green curve in Figure 5 of the main text). Figure S12: Raw fluorescence spectra of SP-L7a hybrid in the presence of LNABc (brown curve in Figure 5 of the main text). Figure S13: Raw fluorescence spectra of SP-L7b hybrid in the presence of LNABc (orange curve in Figure 5 of the main text). Figure S14: Raw fluorescence spectra of SP-L7c hybrid in the presence of LNABb (cyan curve in Figure 5 of the main text). Figure S15: Full concentration-dependent curve for 100 nM SP in the presence of varying concentrations of L7a (0-1000 nM). Fluorescence signal reaches a plateau around 100 nM PM concentration, signifying the expected 1:1 hybridization ratio of SP with L7a target sequence. The linear portion of this curve is shown in Figure 6 of the main text. Figure S16: Raw fluorescence spectra of concentration-dependent curve of Figure S15.
