# Supplementary material for: Detection of Several Homologous MicroRNAs by a Single Smart Probe System Consisting of Linear Nucleic Acid Blockers
Source: Molecules. 2019 Oct 14;24(20):3691. doi: 10.3390/molecules24203691 (PMC6832958; doi:10.3390/molecules24203691)
Supplement: Supplementary file 1 [file molecules-24-03691-s001.pdf]

Supplementary Material for
Detection of Several Homologous MicroRNAs by a Single Smart Probe System Consisting of Linear Nucleic Acid Blockers

Sulayman A. Oladepo* and Basiru O. Yusuf

Department of Chemistry, King Fahd University of Petroleum and Minerals,

Dhahran 31261, Kingdom of Saudi Arabia

* Corresponding author: (voice): +966 13 860 7103; (fax): +966 13 860 4277; (e-mail):

saoladepo@kfupm.edu.sa

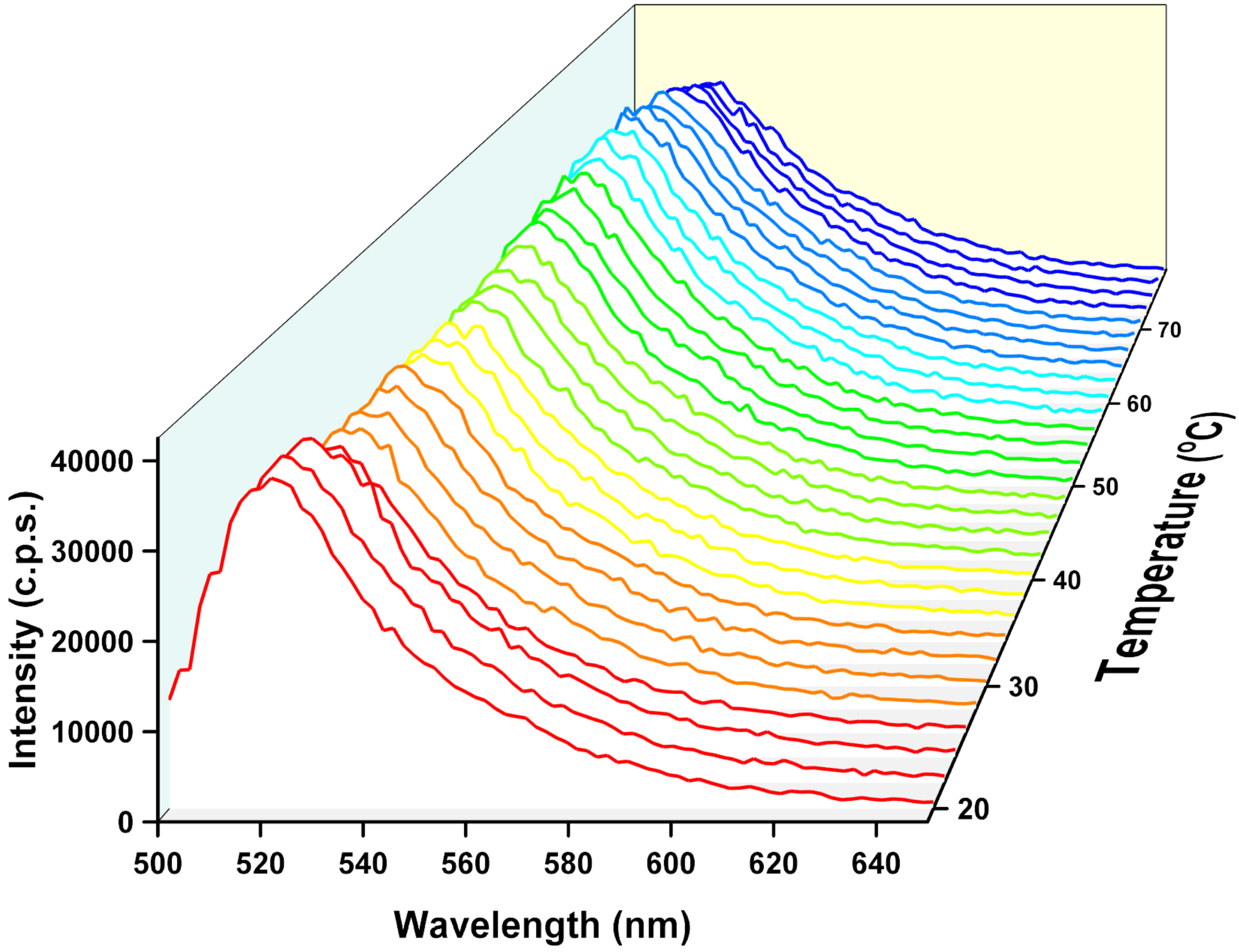

Figure S1. Raw fluorescence spectra of SP only (red curve in Figures 2, 3 and 5 of the main text and Figure S7 below).

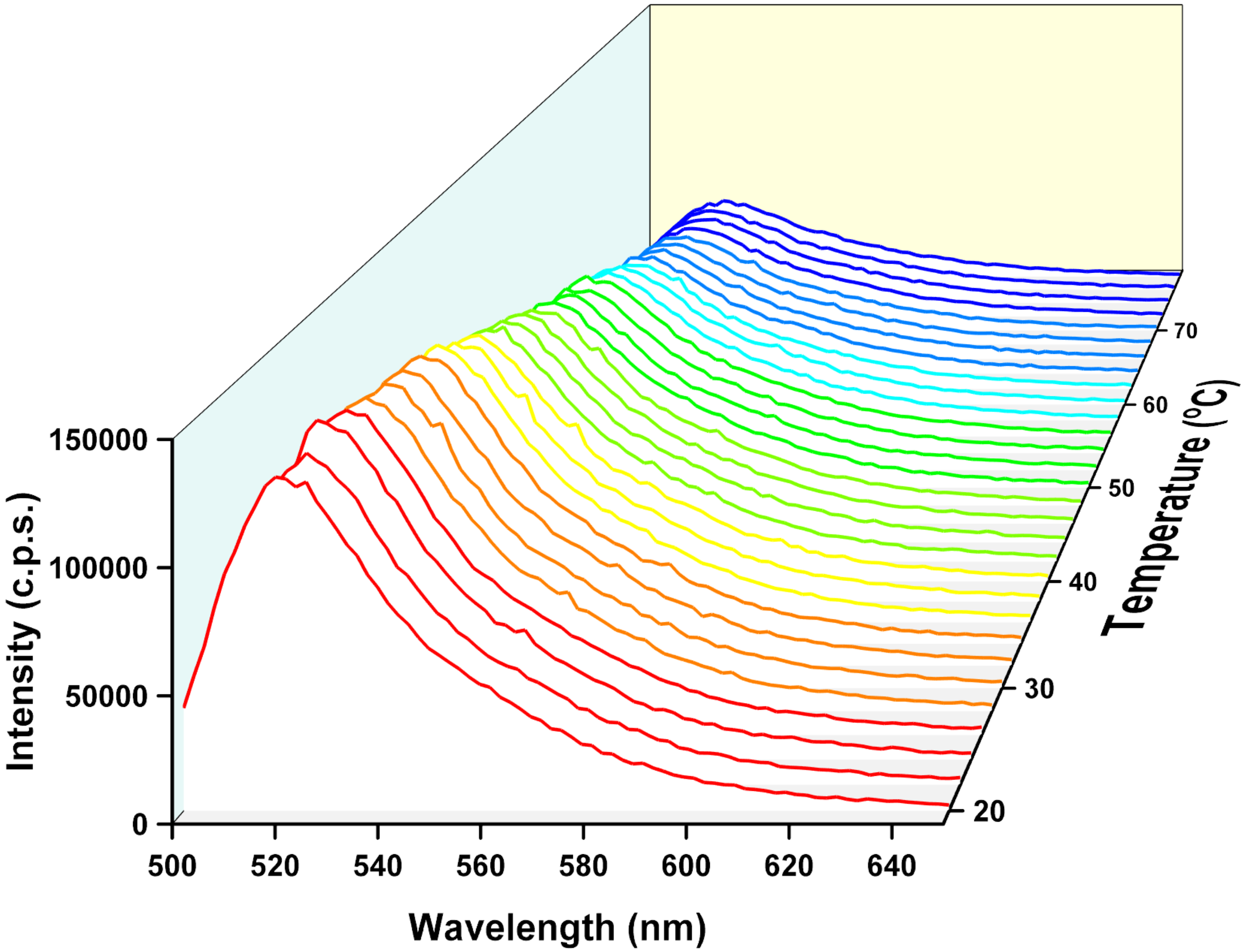

Figure S2. Raw fluorescence spectra of SP-L7a hybrid (black curve in Figures 2, 3 and 5 of the main text).

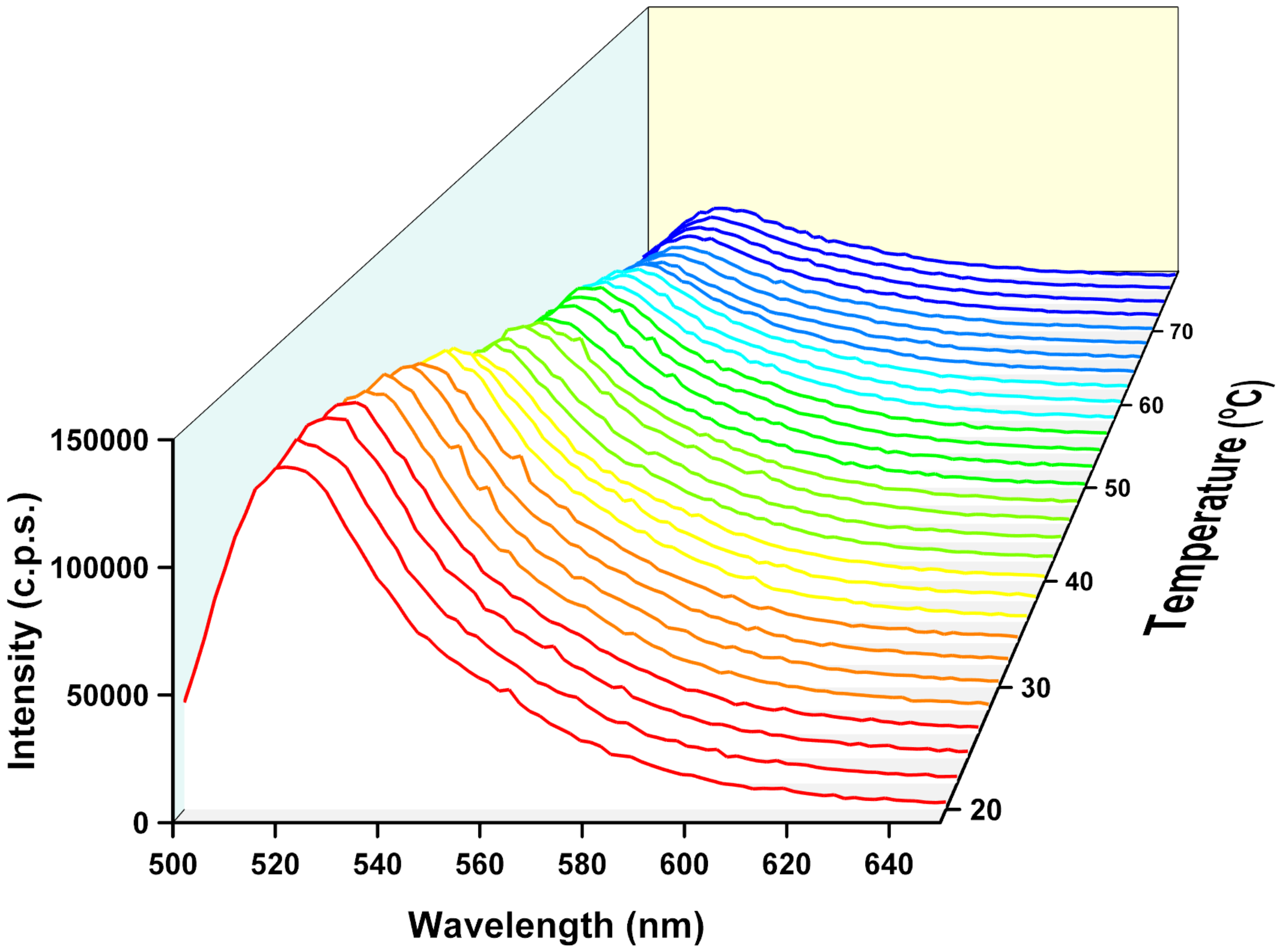

Figure S3. Raw fluorescence spectra of SP-L7b (magenta curve in Figure 3 of the main text).

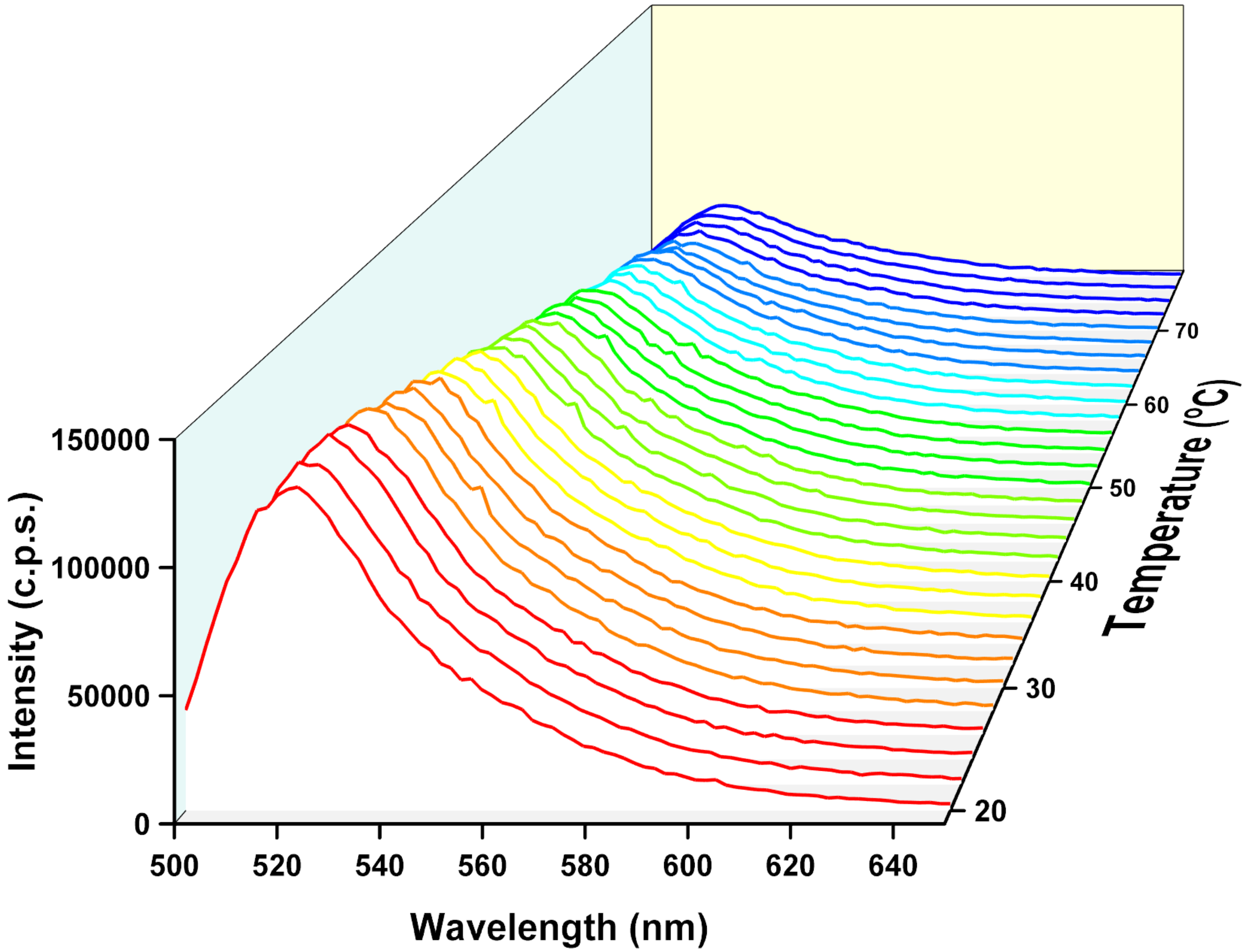

Figure S4. Raw fluorescence spectra of SP-L7c (blue curve in Figure 3 of the main text).

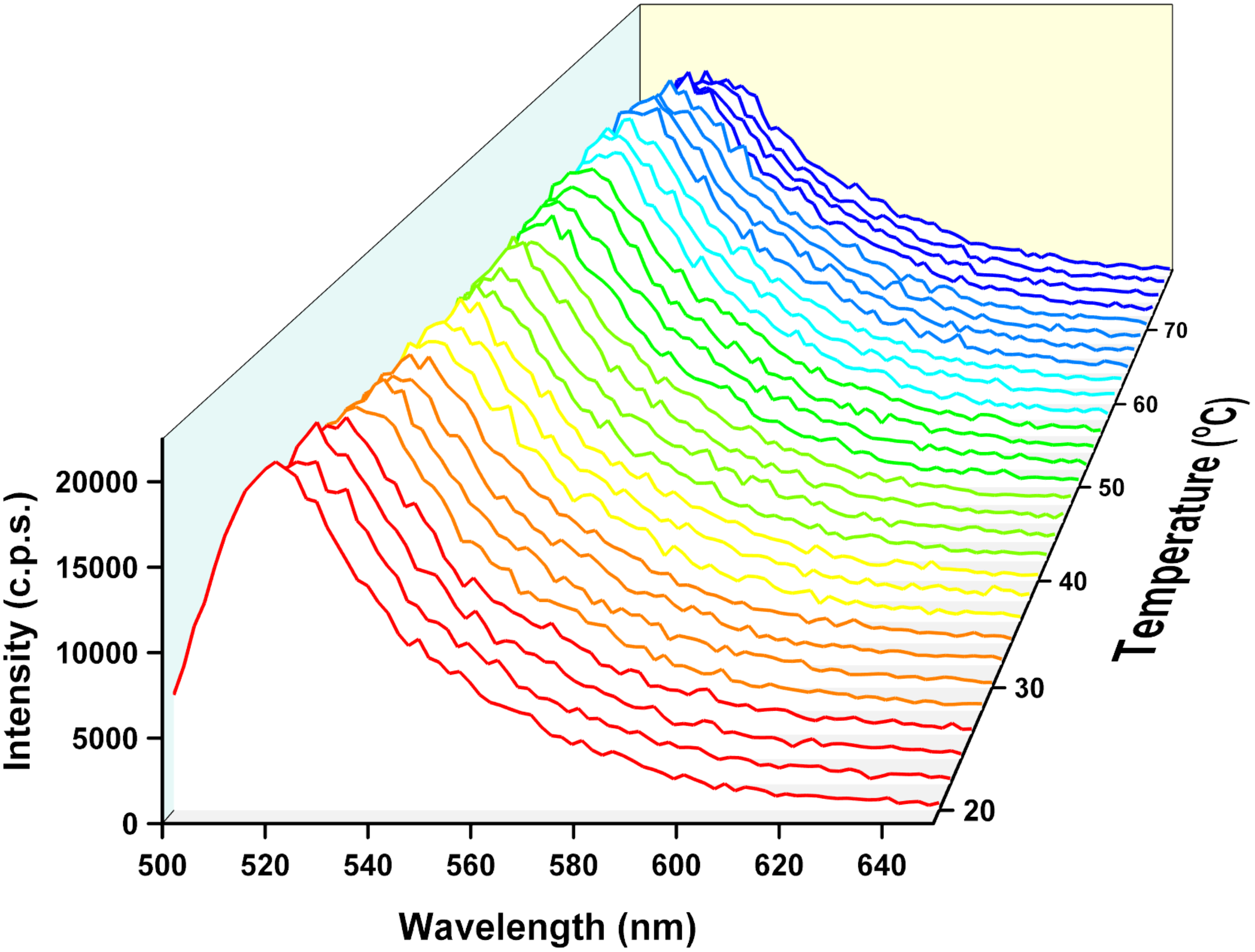

Figure S5. Raw fluorescence spectra of SP/L7b-LNABb (magenta curve in Figure 5 of the main text and Figure S7 below).

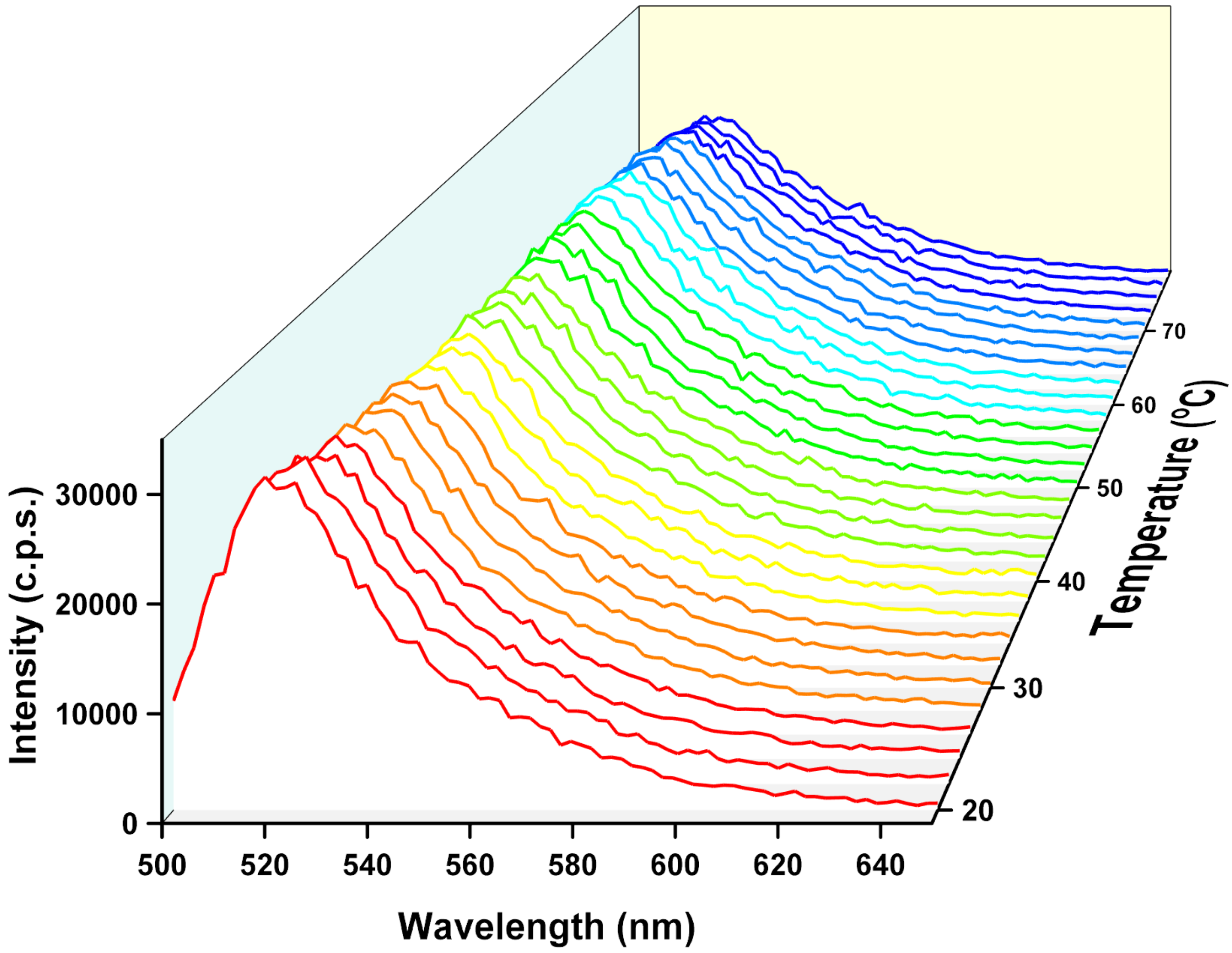

Figure S6. Raw fluorescence spectra of SP/L7c-LNABc (blue curve in Figure 5 of the main text and Figure S7 below).

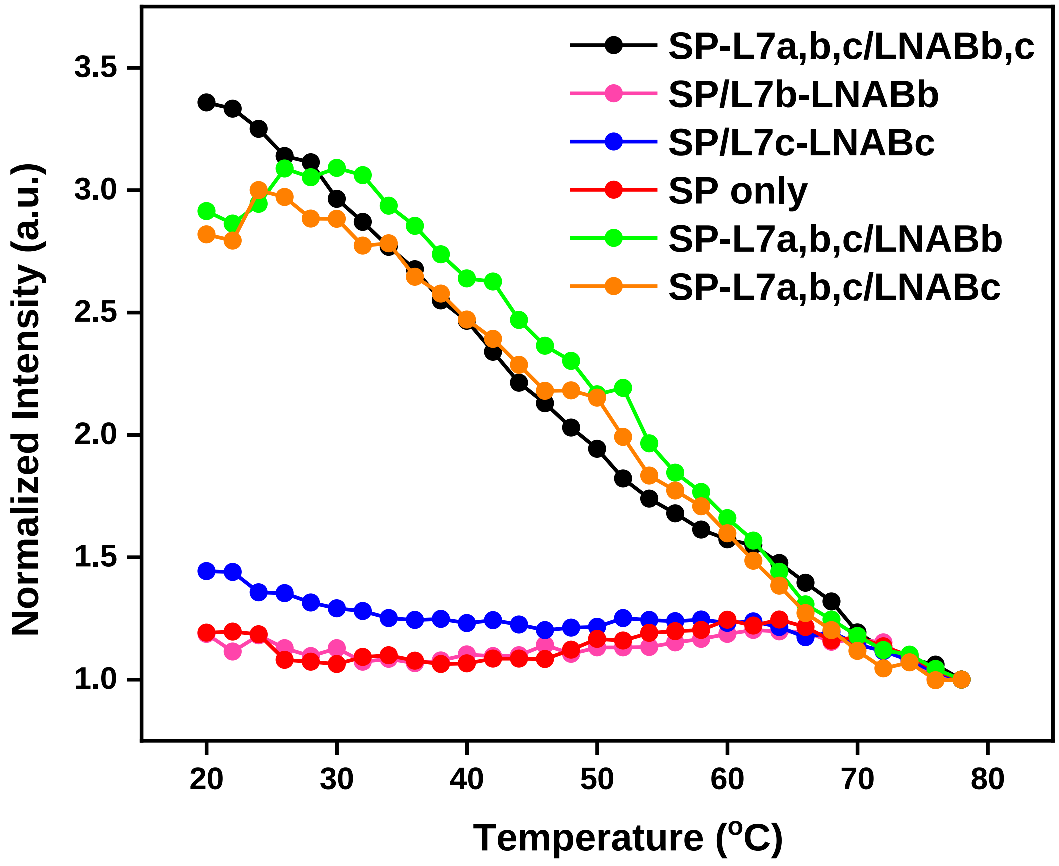

Figure S7. Melting profiles of SP-L7a hybrid in the presence of L7b, L7c, and their corresponding nucleic acid blockers, LNABb and LNABc. SP-L7a,b,c/LNABb,c, represents SP-L7a hybrid in the presence of homologous 'mismatch' sequences L7b and L7c and their respective blockers, NABb and NABc. SP-L7a,b,c/LNABb represents SP-L7a hybrid in the presence of L7b and L7c and NABb, while SP-L7a,b,c/LNABc represents SP-L7a hybrid in the presence of L7b and L7c and NABc. In all cases, the signal intensity of SP-L7a,b,c, is similar to SP-L7a in Figure 2, Figure 3 and Figure 5 of the main text. This suggests little or no hybridization between L7a and NABs, while the signals of SP/L7b-NABb and SP/L7c-NABc hybrids are essentially the same as that of SP alone.

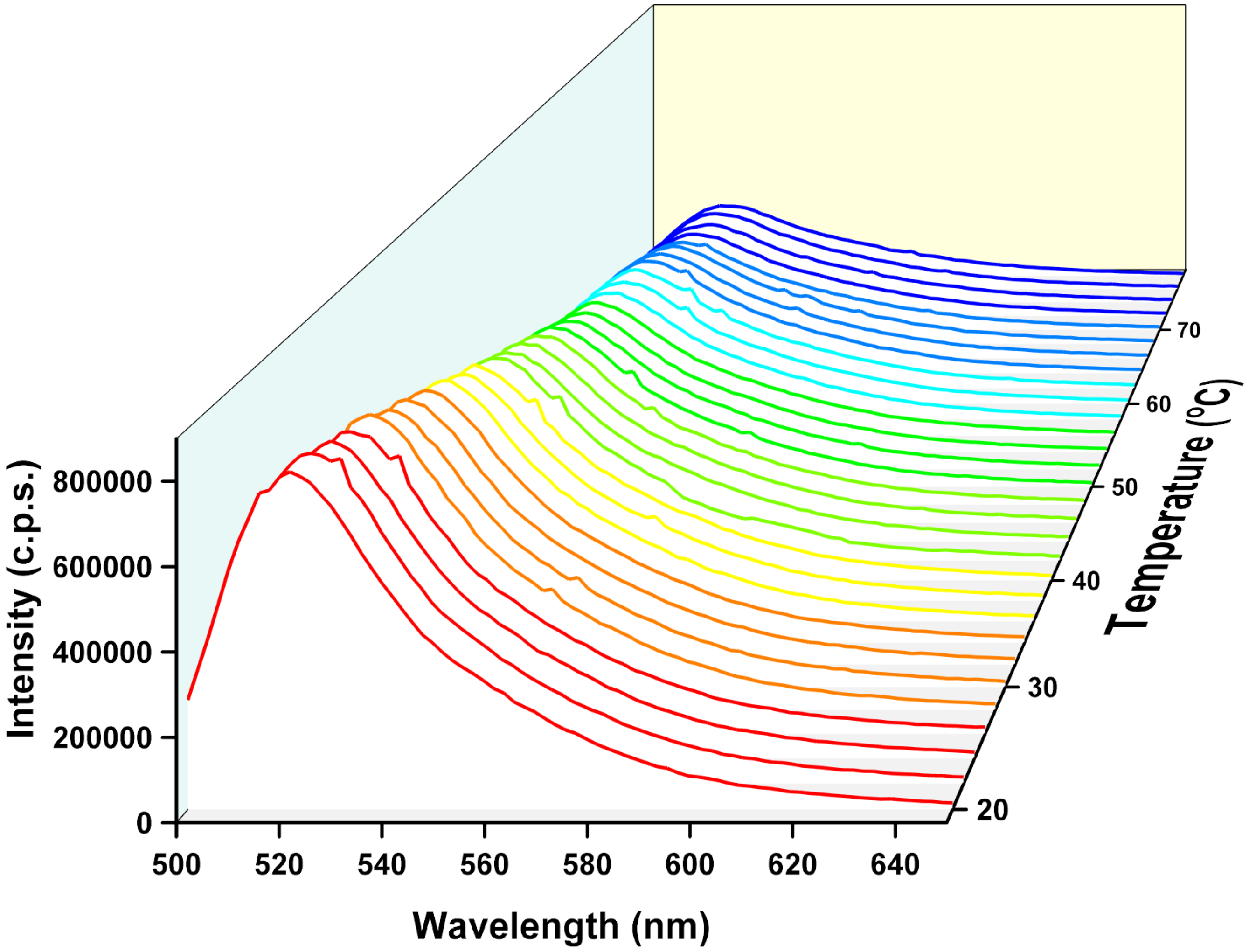

Figure S8. Raw fluorescence spectra of SP-L7a hybrid in the presence of L7b, L7c, and their corresponding nucleic acid blockers, LNABb and LNABc (black curve in Figure S7).

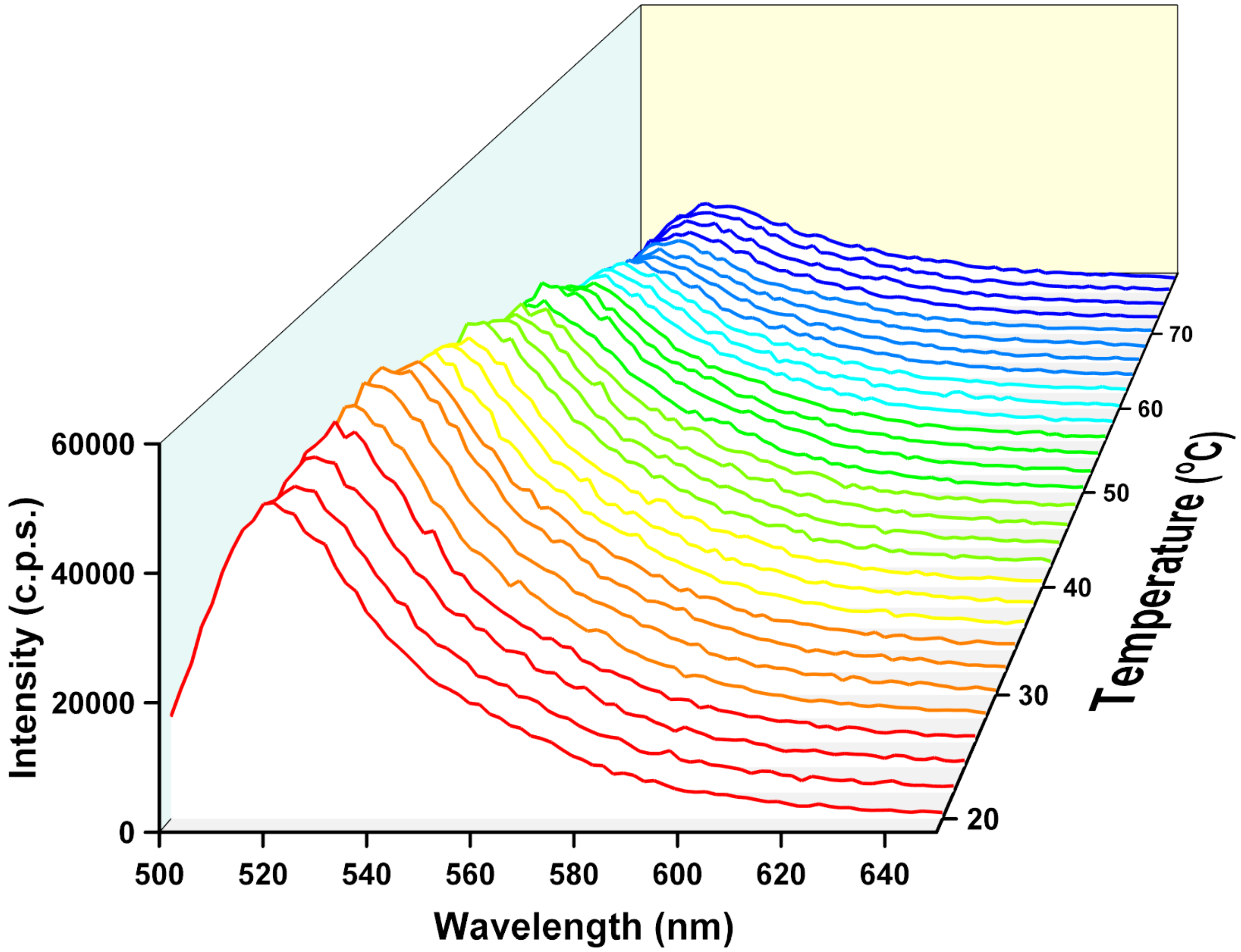

Figure S9. Raw fluorescence spectra of SP-L7a hybrid in the presence of L7b, L7c, and LNABb (green curve in Figure S7).

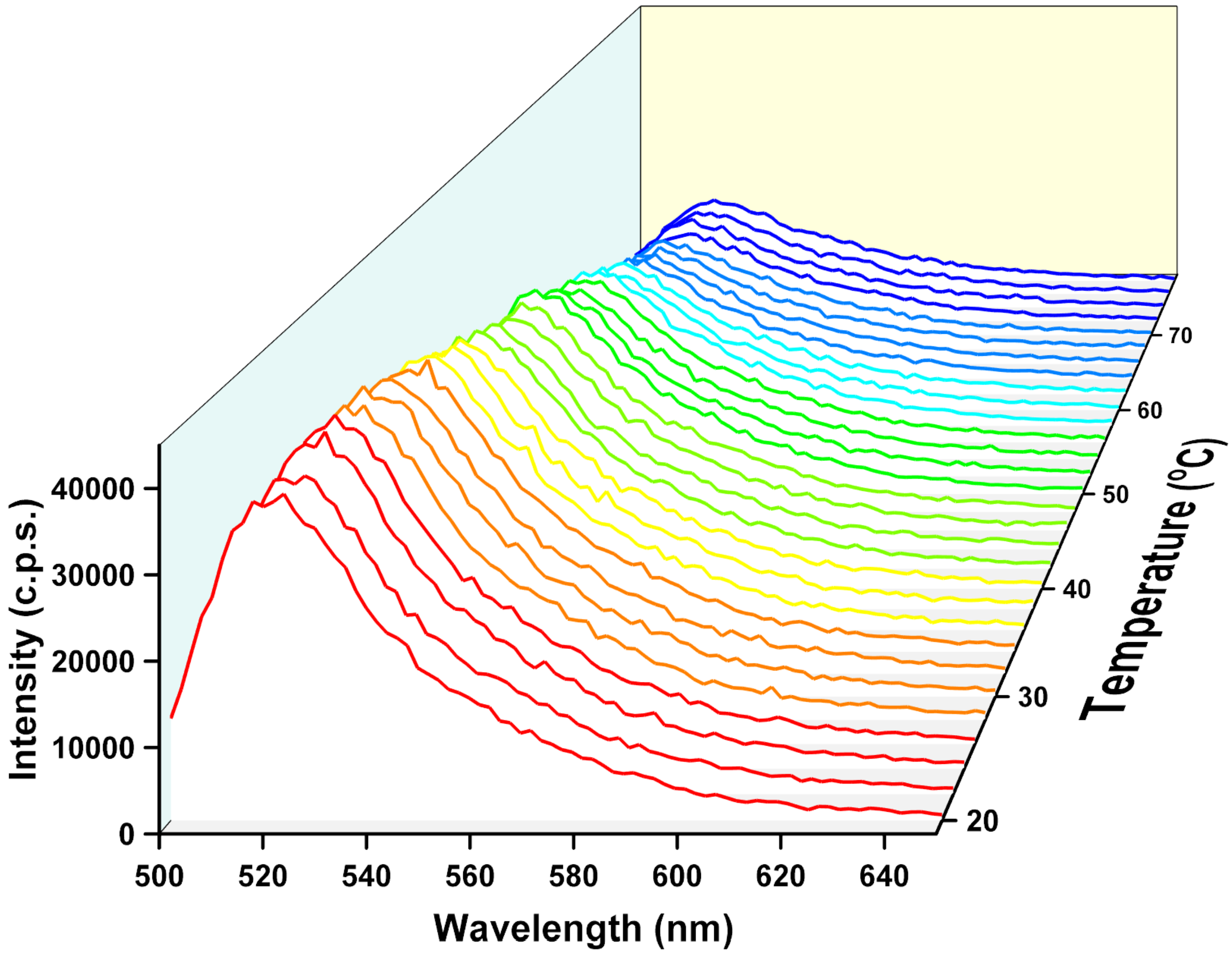

Figure S10. Raw fluorescence spectra of SP-L7a hybrid in the presence of L7b, L7c, and LNABc (orange curve in Figure S7).

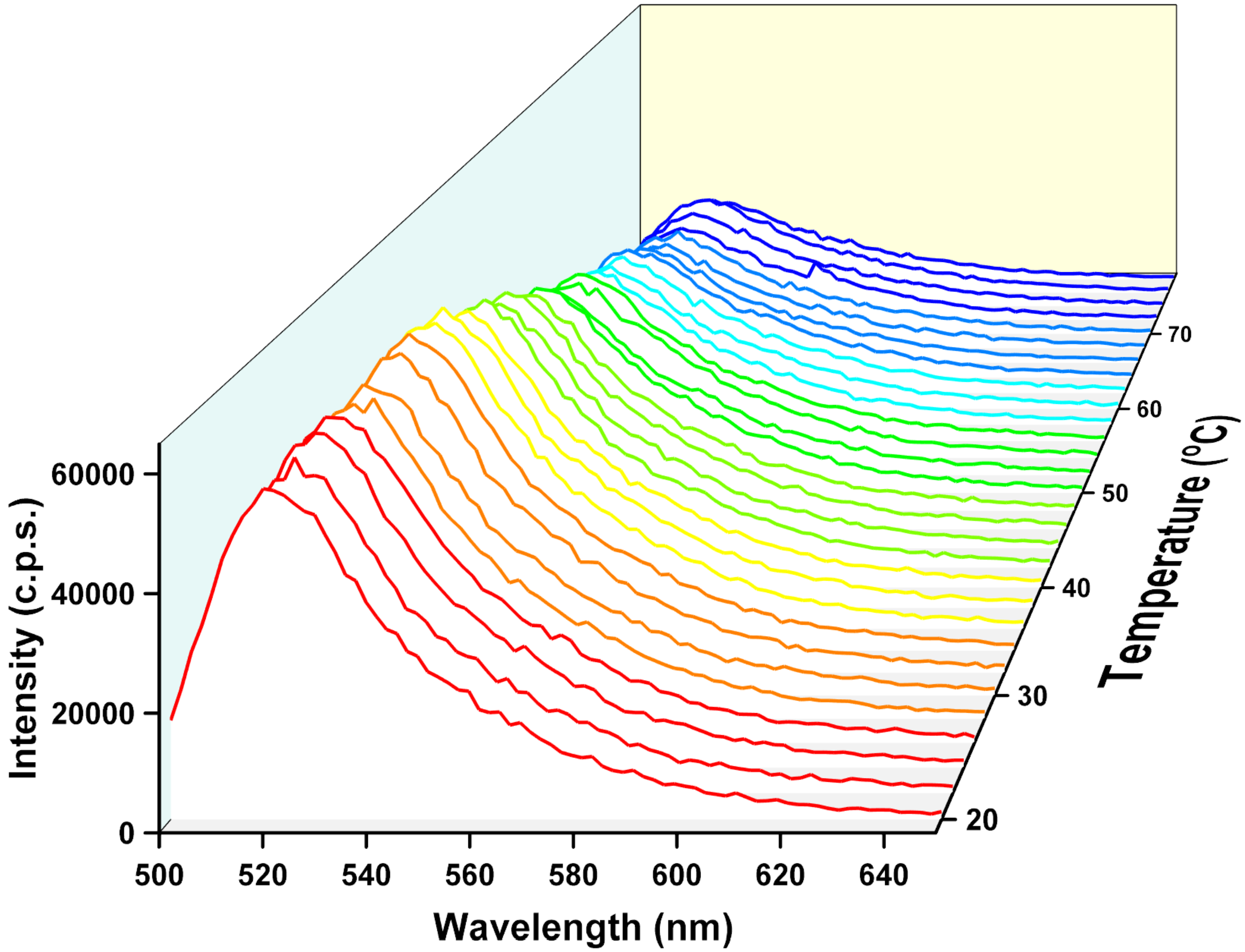

Figure S11. Raw fluorescence spectra of SP-L7a hybrid in the presence of LNABb (green curve in Figure 5 of the main text).

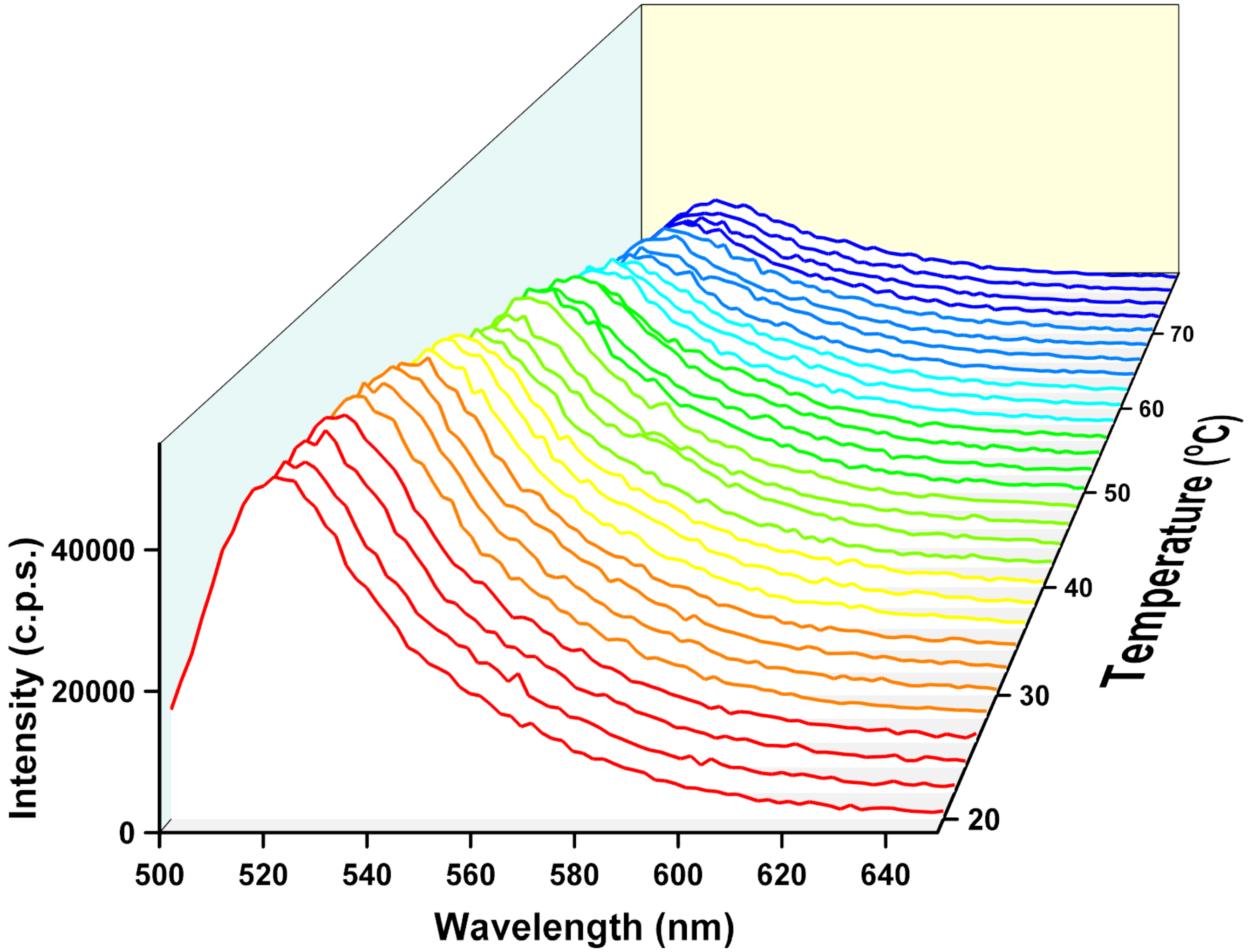

Figure S12. Raw fluorescence spectra of SP-L7a hybrid in the presence of LNABc (brown curve in Figure 5 of the main text).

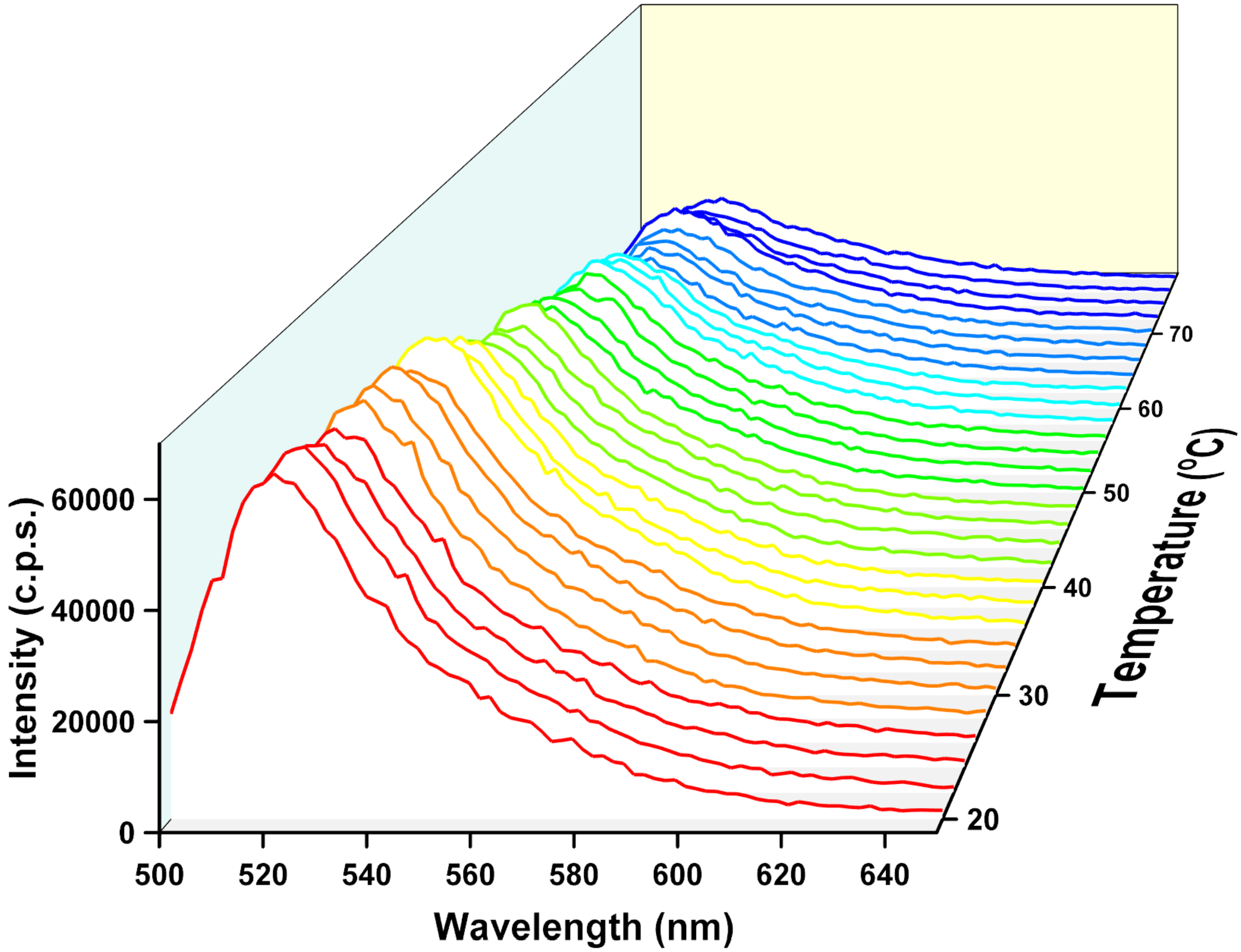

Figure S13. Raw fluorescence spectra of SP-L7b hybrid in the presence of LNABc (orange curve in Figure 5 of the main text).

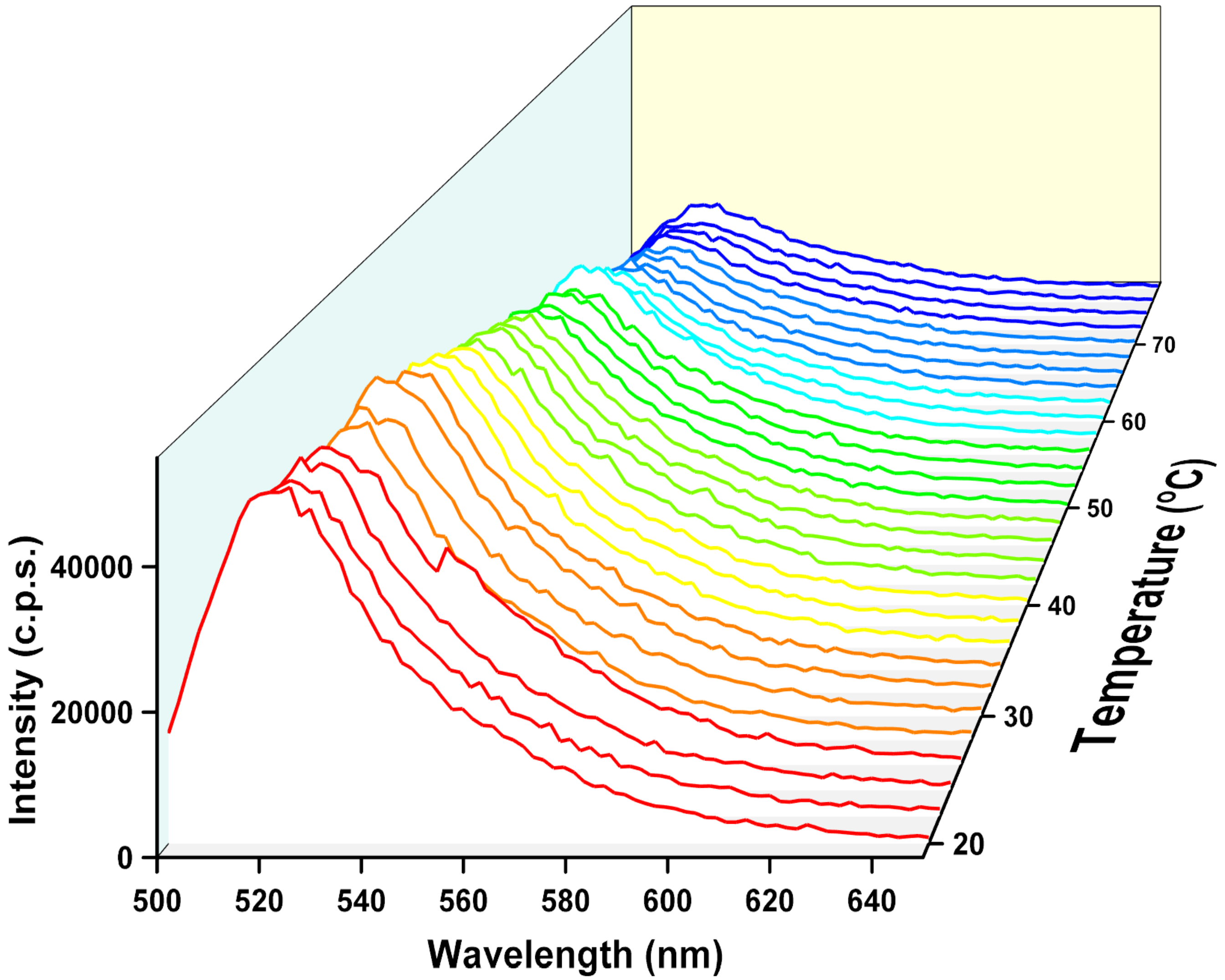

Figure S14. Raw fluorescence spectra of SP-L7c hybrid in the presence of LNABb (cyan curve in Figure 5 of the main text).

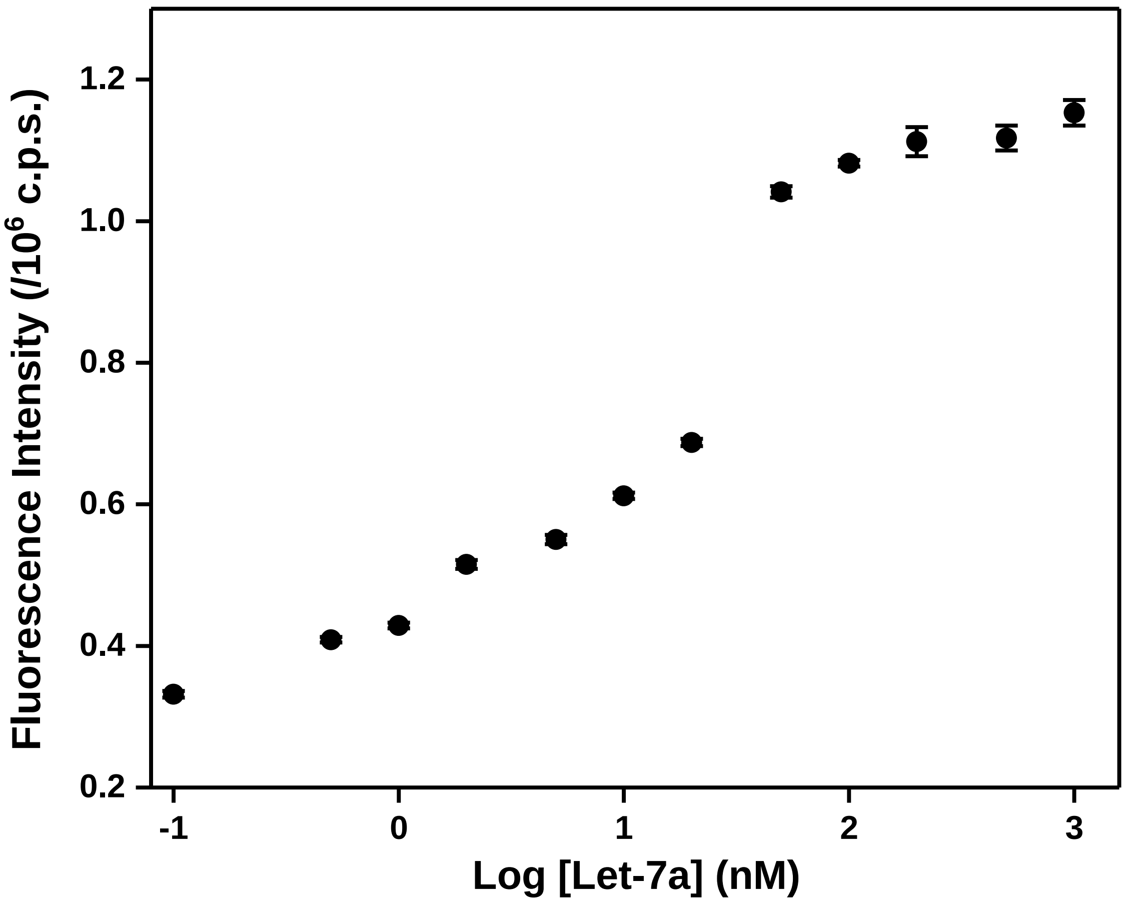

Figure S15. Full concentration-dependent curve for 100 nM SP in the presence of varying concentrations of L7a (0-1000 nM). Fluorescence signal reaches a plateau around 100 nM PM concentration, signifying the expected 1:1 hybridization ratio of SP with L7a target sequence. The linear portion of this curve is shown in Figure 6 of the main text.

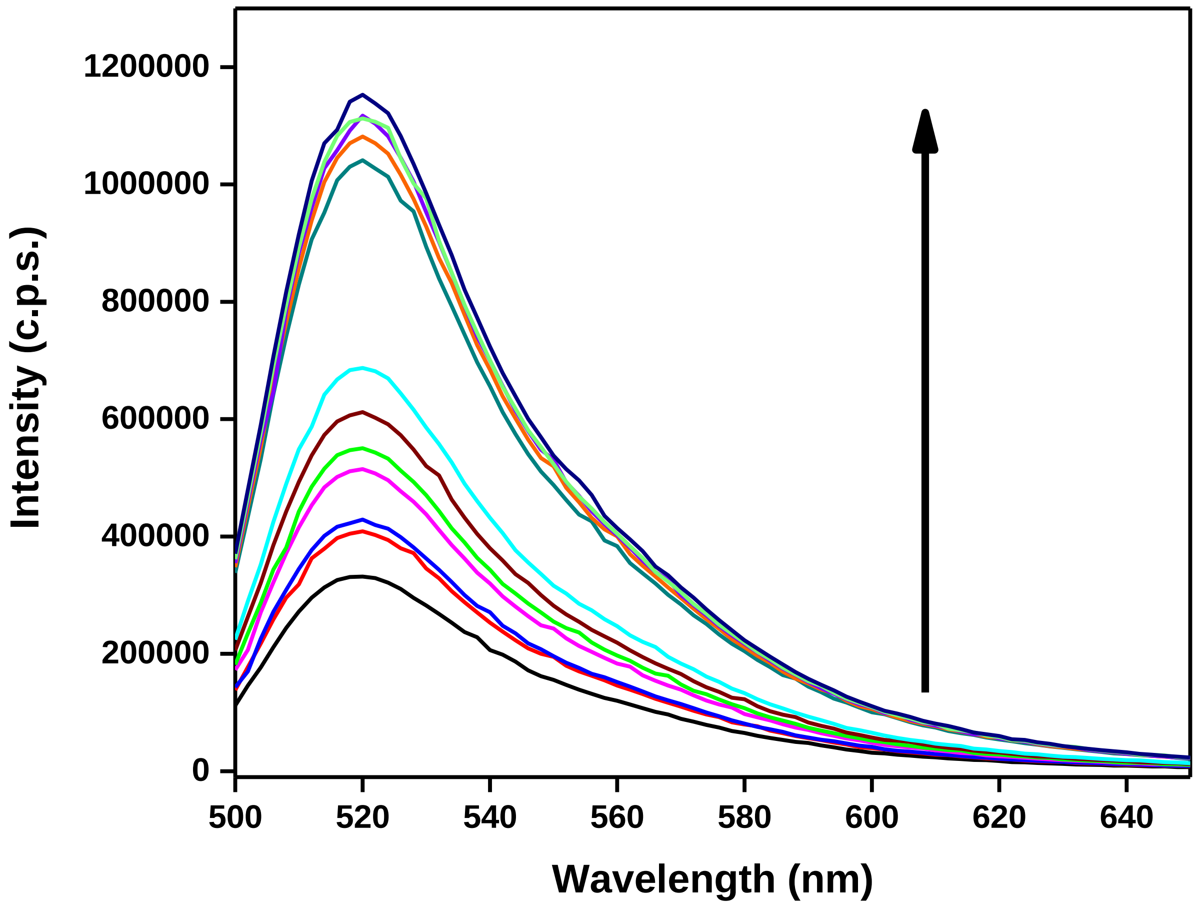

Figure S16. Raw fluorescence spectra of concentration-dependent curve of Figure S15.
